# Peculiarities and Diversified Symptoms Manifested in Hysterical Subjects

**Published:** 1856-05

**Authors:** J. G. Westmoreland

**Affiliations:** Atlanta, Ga.


### ARTICLE IV.

*Peculiarities and diversified symptoms manifested in Hysterical Subjects.* By J. G. Westmoreland, M. D., Atlanta, Ga.

So deceptive are some of the violent symptoms of this disease, that a very general impression exists among the people, that the entire dependence of the affection is upon feigned distress. This is so common, that but few cases can, with propriety, be reported to the patient or friends under their true name. The physician is constantly taxed with the inconvenience of inventing some term, showing the nature of the derangement, by which to assuage the anxiety of friends, and at the same time avoid what is considered equivalent to an accusation of dissembling on the part of the patient.

In hysteria, the derangement, for the most part, is functional, depending on perversion or debility of the nervous energies. Sensation alone is sometimes at fault. Globus hystericus, or the sensation of approaching asphyxia, is one of the most common attendants upon the disease in its most simple form. Symptoms of the various organic lesions are so nearly similated, without the slightest disease of the structure, that the unfortunate sufferer is not unfrequently unpbraided with entertaining hypochondriacal or imaginary sensations and fears. All this arises from a want, on the part of observers, of a just understanding of the functions of the nervous system, and the characteristics of its perversion. It is a fact, too familiar to be repeated, that the whole organic system is dependent for its natural functions upon a proper nervous influence, and that a modification or perversion of the latter, will make a corresponding modification of the former.

So it will be seen, that a perversion of the functions of an organ may exist as perfectly from this cause as though organic structural lesion existed; and unfavorable prognosis, predicated on a misconception of the pathological condition, as has come to my knowledge, is very likely to result.

In order properly to understand the nature and tendencies of nervous diseases, certain physiological facts must be borne in mind. It is known that some organs, through the nervous distribution, are so intimately connected with each other, and with the general nervous system, that, from a radical disturbance in one, a sympathetic derangement is set up in all so connected.

According to this general sympathetic influence, any irritating ingesta causes the most intense cephalalgia, while comparatively slight effect is discovered in the stomach itself. The same sympathy exists with the skin and alimentary canal. When great heat and comparative dryness of the latter prevail, a like modification of the cutaneous transpiration is found to occur. Hence, iced drinks are the best diaphoretics in such cases.

Not only in these particulars do sympathetic influences operate, but the cerebro-spinal axis itself is sometimes the seat of irritation by means of the nerves supplying an organ laboring under derangement. An example of this form of sympathetic action is found in that condition of the encephalon which often occurs in infantile gastro-enteritis. Perhaps a majority of children who die from this disease, have unmistakable evidences of cerebral oppression.

The impression thus made on the nervous centres in the vicinity of the nerve conveying the impression, so deranges their functions, that impressions, carried by other nerves supplying different organs, are abnormal.

This brings us to the consideration of the true pathology of hysteria. As its name implies, the ancients considered it to depend upon uterine disturbance. And although nothing but the difficulty of changing a name so long and so generally adopted, prevented its being expunged from the catalogue of diseases, owing to the prevailing views of its pathology; yet the careful investigation of the uterine functions in hysterical females will, in our humble opinion, eventually satisfy all who take the trouble to do so, that the moderns would scarcely adopt a more appropriate name.

Most, if not all, cases of hysteria occur in subjects of irritation or functional derangement of the womb.

It is not to be denied, however, that symptoms very much resembling this disease, may sometimes be found even in a male, but the symptoms, taken all together, are different. A case very familiar to me, which has engaged my attention, occasionally, for two years past, has something the appearance of the disease under consideration, and arose from indigestion, most probably. In this case, the nerves supplying the intercostal muscles, and perhaps the diaphragm, are at fault. Sometimes the most distressing sense of suffocation comes on without any apparent organic lesion. A feeling of inability to expel the air from the lungs, and general prostration of the nervous energies, are the most prominent symptoms in the attacks. There is evidently derangement of the digestive organs primarily, and it is but rational to conclude that, as the stomach holds a prominent position as the great center of nervous sympathies, its derangement is the prime cause of the paroxysms. is in this way that uterine derangement produces that train of peculiar nervous symptoms denominated hysteria. It seems, however, to require, in order to its development, the necessary constitutional peculiarity which every female does not possess; for, in some, almost every variety of uterine disturbance may occur—functional, organic and mechanical—without one symptom ot this peculiar disease, whereas, in a hysterical constitution, a comparatively slight cause in this organ will produce the whole catalogue of the usual effects. It is remarkable with what indifference some females regard prolapsus uteri, when even amounting to procidentia, while others cannot retain the upright position with the slightest descent.

Whether it is from having become accustomed to the displacement, or from a difference in the susceptibility of the nervous system, it is not certain. I am, however, inclined to the latter opinion.

One woman will show no evidences of nervous derangement, from chronic metritis, dysmenorhæa, &c., while another will be thrown into a paroxysm of hysterics from the mildest of these disturbances.

Hysteria is generally readily recognized by the experienced practitioner, but from its resemblance to other diseases, it is not so easily detected by the young physician. Various dangerous diseases are so nearly imitated, that error in diagnosis occurs sometimes with the most experienced. Paralysis, epilepsy, asthma, laryngitis, pleurisy, hepatitis, peritonitis, intussusception and gastritis, are some of the diseases for which it may be mistaken. I recollect distinctly, that the first case I ever attended so nearly similated disease of the brain, that physician and friends were alarmed at the prospects of recovery.

Another case of recent date, that is fresh in my memory, of obstinate constipation in a hysterical woman, so far exceeded any thing I had before witnessed, that, knowing as I did the constitutional tendency, I could but sometimes fear intersusception. The case was attended by myself and brother for seven or eight days after the patient, a negro woman 40 years old, had been confined to bed; and her report, which her owner did not doubt, was, that it had, at our first visit, been three weeks since her bowels were evacuated. As to the time that had elapsed previous to our first visit, of course, we can only rely on the statement of the patient, but being confident that no fœces passed during the week we attended her, and finding so little alteration in her symptoms during that time, we are disposed to credit her statement. Active cathartics and saline enemata were repeatedly used without any effect whatever. Opiates and antispasmodics were used with the view of allaying the pain, which was sometimes distressing, and to counteract any irregular contractions of the bowel that might exist. Finally, the lower bowels were filled with a solution of common salt, by the pump, but did not then produce any effect until being filled in this way, was retained by pressure for thirty or forty minutes, when, after causing severe pain, the desired effect was produced.

There is a singular fact, and one that should be borne in mind, connected with the history of hysterical patients, and that is, that the arterial circulation is rarely disturbed.

When it would seem from most of the symptoms that life was almost extinct, the action of the heart is as calm and natural as in the most perfect health. With the acute tenderness found in the most violent peritonitis, where it is expected to find the pulse corded, and numbering a hundred and twenty or thirty beats, very little variation from the healthy standard is observed.

This is generally the case, when not complicated with any local or general disease, calculated in itself to excite febrile reaction, but at the same time hysterical symptoms may present themselves in other diseases; and even the derangement which we have alledged as the cause of this condition of the nervous system, may amount to a perfect inflammatory condition, such as will inevitably derange the circulatory system. When this is the case, however, a name should be applied denoting the pathological condition, as hysteritis, &c.

